# Synthesis of antibacterial poly(*o*-chloroaniline)/chromium hybrid composites with enhanced electrical conductivity

**DOI:** 10.1186/s13065-018-0416-3

**Published:** 2018-04-26

**Authors:** Mirza Nadeem Ahmad, Fakher Rafique, Faisal Nawaz, Tahir Farooq, Muhammad Naveed Anjum, Tajamal Hussain, Sajjad Hassan, Madeeha Batool, Hamad Khalid, Khurram Shehzad

**Affiliations:** 1 Department of Applied Chemistry, Government College University, Faisalabad, 38030 Pakistan; 2 University of Engineering & Technology, Lahore, 54000 Pakistan; 3 Institute of Chemistry, University of the Punjab, Lahore, 54000 Pakistan; 4 Interdisciplinary Research Center in Biomedical Materials, COMSATS Institute of Information Technology, Lahore, 54000 Pakistan; 5 Department of Information Technology and Electronics, Zhejiang University, Hangzhou, 310027 China

**Keywords:** Polyorthochloroaniline, Chromium nanoparticles, Conductivity, Nanofiller, Nanocomposite

## Abstract

Electrically conductive polyorthochloroaniline/chromium nanocomposites (POC/Cr NCs) were prepared by in situ chemical oxidative polymerization of orthochloroaniline in the presence of Cr nanoparticles (Cr NPs). The load percentage of Cr nanofiller was varied in POC matrix to investigate the effect of Cr nanoparticles on the properties of the nanocomposites. The composition, structure, and morphology of POC and its composites were examined by Scanning electron microscopy, Fourier transform infrared spectroscopy, and UV–visible spectroscopic analysis. The antibacterial potential of POC and its composites was evaluated by the disc diffusion method against *Escherichia coli* and *Bacillus subtilis*. The results showed the improved antibacterial potential with the increase in the load percentage of nanofiller. The electrical conductivity of polymer and its composites was measured and correlated with the load percentage. The results showed that electrical conductivity of the composites was enhanced with the increase in load percentage of Cr nanoparticles.
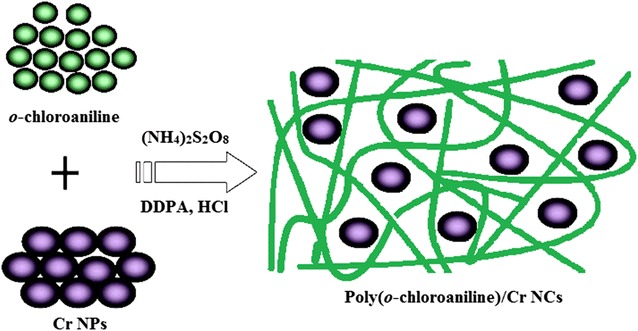

## Introduction

Conductive polymers are a class of synthetic metals which combine chemical and mechanical properties of polymers with electronic properties of semiconductors and metals [1, 2]. Typical conducting polymers include polyacetylene, polyaniline, polypyrrole, polythiophene, poly(*p*-phenylene), poly(phenylenevinylene), and polyfuran [3]. Ease of synthesis, ecological stability, easy transformation to cathodic protection, and distinct electronic, magnetic, biological and optical properties have attracted researchers to focus on polyaniline and its derivatives [4]. Organic–inorganic composite materials have attracted considerable attention because they can combine the advantages of both components and may provide special properties through synergistic effects [5]. To advance and expand the scope of the conductive organic materials, inorganic materials like metals and metal oxides have been incorporated to form highly functionalized materials for various applications in the fields of electronics, sensors, catalysis, energy, electromagnetic interference shielding and biomedicine [6, 7]. The nanoscale inorganic fillers have a high surface to volume ratio and therefore are expected to modify the biological, optical, electrical, thermal and dielectric polymers drastically [8]. Recently, metals and metal oxide particles have been incorporated in the conductive polymers to form nanocomposite (NCs). NCs show combination of properties such as conductivity, catalytic, electrochemical, optical and antimicrobial properties [9]. Chemically synthesized nanoparticles have attracted much attention because of the unique properties associated with their magnitude of size and of its uniform distribution [10]. Because of their small size, nanoparticles have properties of advanced materials that are significantly different from those of their bulk counterparts [11]. Therefore, the addition of nanofiller to an inherently conductive polymer could produce the materials with an even high level of conductivity [12–20]. Recently, the demand for conducting plus antibacterial materials has increased manifold due to their tunable properties which makes them suitable for different areas of application.

In the present study, we report the preparation of chloro-functionalized PANI, i.e. poly(*o*-chloroaniline). POC was further composited with Cr nanofiller to synthesize the materials with enhanced electrical conductivity and antibacterial action. Oxidative polymerization technique was employed for in situ syntheses of POC/Cr NCs by using ammonium persulfate as an oxidizing agent in the presence of Cr nanofiller and 4,4′-diaminodiphenylamine. Furthermore, we have studied the antibacterial and electrical properties of the composite materials and effect of nanofiller load percentage.

## Materials and methods

### Materials

Orthochloroaniline, 4,4′-diaminodiphenylamine (DDPA) and ammonium persulfate (APS) were procured from Sigma-Aldrich (Germany). The chromium nanoparticles were purchased from GNM Corporation France and utilized without any treatment. All the chemicals and reagents were of analytical grade and used as received.

### Synthesis of poly(*o*-chloroaniline)

**Fig. 1:**
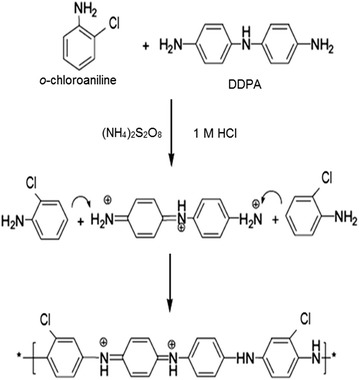
Schematic illustration of polymerization of *o*-chloroaniline

Polyorthochloroaniline was polymerized by acid catalyzed oxidative polymerization of *o*-chloroaniline. Specifically, 5 g of *o*-chloroaniline (monomer) and 0.5 g of 4,4′-diaminodiphenylamine were dissolved in 20 ml of 1 M HCl to prepare solution A. DDPA was added 10% weight of the monomer. The temperature of solution A was maintained at 4 °C in an ice bath. The solution B was prepared by dissolving 5 g of ammonium persulfate in 20 ml of 1 M HCl. The monomer to APS mass ratio was kept as 1:1. Solution A was kept on magnetic stirring in an ice bath and solution B was then added drop wise into solution A for 3 h. The color of the solution was turned green which indicated the polymerization process. The stirring was stopped and the solution was kept undisturbed for 24 h. Then, the solution was centrifuged at 4000 RPM and washed first with double distilled and methanol at the last. Finally, the product was dried in a vacuum oven at 100 °C for 1 h. The de-doping and re-doping were performed using 1 M HCl, and then with 1 M NH_4_OH. After de-doping, the color was turned from green to blue and it was reverted to its original color after re-doping with acid [21] (Fig. 1).

### Synthesis of poly(*o*-chloroaniline)/chromium nanocomposites

Nanocomposites of POC and Cr NPs were prepared by following the similar procedure as employed for the polymerization of *o*-chloroaniline. According to the desired weight percentage, the Cr NPs were added to the monomer at the time of polymerization. The *o*-chloroaniline along with DDPA was dissolved in 1 M HCl and then Cr NPs were added to this solution. The contents were sonicated for 15 min to prepare the uniform dispersion. After sonication, the mixture was stirred continuously via magnetic stirrer and APS solution was added drop wise as an oxidizing agent. The temperature was maintained at 4 °C during the reaction. By following the above recipe, all other composites were prepared by varying the Cr NPs load percentage (1, 3, 5, 7 and 10%). After adding the APS, the solution was kept on stirring for 3 h. The formation of green color showed the preparation of the product. Finally, the solution was centrifuged and washed with water and methanol. The products were then dried in a vacuum oven at 100 °C for 3 h and proceeded for characterization.

### Characterization

Various analytical tools were used to characterize the structure, morphology, and properties of POC and POC/Cr nanocomposites. UV–visible spectra were obtained from UV–visible spectrophotometer (Varian Cary 50) in the scanning range of 200–800 nm at the scanning rate of 400 nm/min. The baseline line correction was done by distilled water as a blank reference. FTIR spectra were recorded from FTIR spectrometer (Impact 400, Nicolet, Waltham, MA) in the range of 400–4000 cm^−1^ by KBr pellet method. SEM images were obtained from Hitachi S-4700 operating at 30 kV. The antibacterial potential was evaluated by the agar disc diffusion method. The electrical conductivity was recorded by using CyberScan PC 510 conductivity meter. The conductivity of the dispersion was measured containing 2 mg of sample in 1 ml of deionized water.

## Results and discussion

Polyorthochloroaniline/chromium nanocomposites were characterized by using FTIR, UV–visible, SEM and electrical conductivity measurements.

### FTIR analysis of POC and POC/Cr NCs

Polyorthochloroaniline/chromium nanocomposites were analyzed using FTIR technique. It is employed to determine the different vibrational modes shown by the polymer and its composites. The FTIR spectra of polymer and polymer nanocomposites were recorded and positions of the absorption peaks were identified. The prominent bands appeared at 1201 and 1499 cm^−1^ due to non-symmetric stretching vibrations of C-6 rings. The infrared spectrum of POC derivatives showed the bands around 955, 875, 785, 720 and 655 cm^−1^ which were assigned to ortho-substituted aryl rings. The signal at 3075 cm^−1^ was evident for stretching vibrations of –NH group present in the polymer. Strong peaks at 1580 and 1515 cm^−1^ were due to benzene rings and quinonoid structure present in the polymer. The stretching vibrational signal of aromatic –CN was appeared at 1305 cm^−1^. The important two peaks were recorded at 918 and 833 cm^−1^ and were assigned to out-of-plane bending vibrations of –CH in 1,2,4-trisubstituted aromatic rings. The chloro group, at the ortho position on the phenyl ring, was indicated by a signal at 750 cm^−1^. All the signals confirmed the synthesis of the polymer and its composites. On the addition of Cr nanofiller, the bands between 3710 and 3510 cm^−1^ were shifted towards lower values and became deeper and wider. This absorption behavior showed the presence of nanofiller in the polymer matrix. In the spectra of POC/Cr NCs, the –NH stretching frequency peak appeared at 3385 cm^−1^, while it was present at 3410 cm^−1^ in the case of POC. The characteristic peaks of quinonoid and benzenoid rings of poly(*o*-chloroaniline) were located at 1580 and 1505 cm^−1^ and aromatic –CN stretching mode appeared at 1300 cm^−1^ [21, 22].

**Fig. 2:**
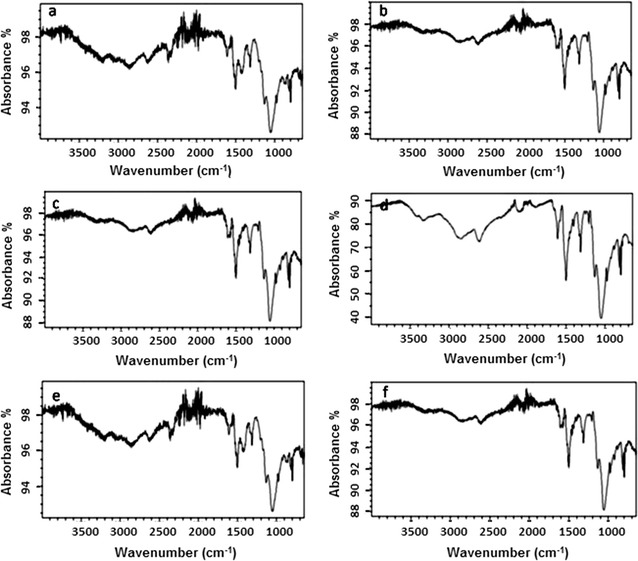
FTIR spectra of **a** POC, and POC/Cr NCs containing **b** 1% Ni NPs, **c** 3% Ni NPs, **d** 5% Ni NPs, **e** 7% Ni NPs, **f** 10% Ni NPs

The bands at 895 and 825 cm^−1^ showed in-plane bending vibrations of –CH present in 1,2,4-trisubstituted aromatic rings. In POC, these bands were located at 920 and 835 cm^−1^. The peak due to chloro group attached to the phenyl ring transition shifted to 755 cm^−1^. When all the peaks of the POC/Cr NCs were compared with POC, it was noted that all the special stretching frequencies were shifted towards the lower frequency. This shift was attributed to the addition of the nanofiller. The FTIR studies confirmed the structural feature of the materials synthesized. The results of FTIR analysis for POC/Cr NCs containing other load percentages were also on the same pattern [23, 24] (Fig. 2).

### UV–VIS absorption of POC and POC/Cr NCs

UV–visible absorptions POC/Cr NCs were measured on UV–visible spectrophotometer in the range of 200–800 nm using distilled water as reference solvent. The prominent band was recorded at 252 nm. The absorption band at 252 nm was evident for π–π* transitions associated with benzene rings and others were due to n–π* transitions of quinonoid rings in the range of 317–320 nm. With the increase in the load percentage of Cr nanofiller, there was a gradual increase in the intensity of these bands. The absorptions of POC/Cr NCs were shifted towards lower wavelengths compared with POC. The blue shift indicated the embedding of Cr nanoparticles in the POC matrix [23, 24].

**Fig. 3:**
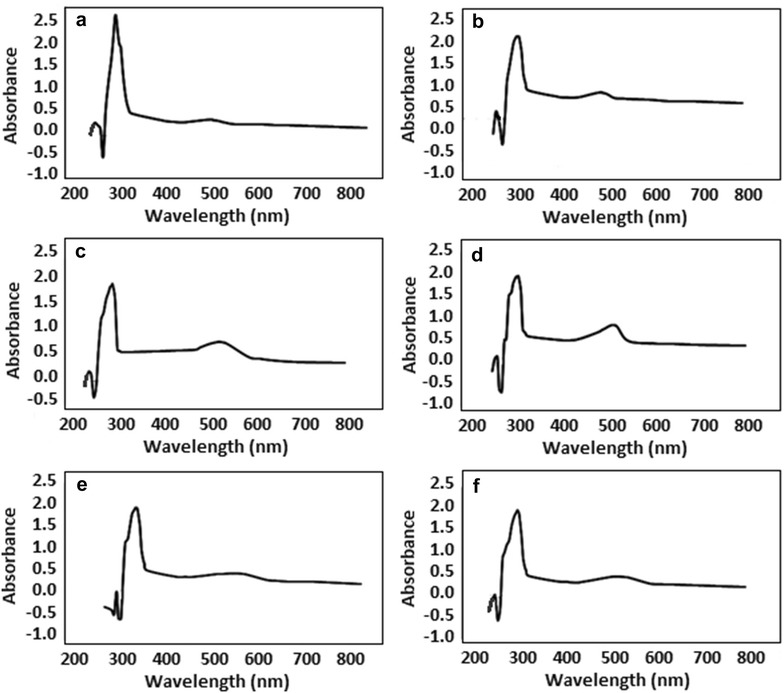
UV–Visible spectra of **a** POC, and POC/Cr NCs containing **b** 1% Ni NPs, **c** 3% Ni NPs, **d** 5% Ni NPs, **e** 7% Ni NPs, **f** 10% Ni NPs

Due to the addition of Cr nanofiller, the n–π* absorption band became slightly wider while it was sharper in case of POC. This phenomenon showed some physical interaction between the polymer matrix and Cr nanoparticles. POC/Cr NC (1%) showed the characteristic band at 320 nm, POC/Cr NC (7%) at 318 nm and POC/Cr NC (10%) at 317 nm. The results demonstrated the good absorption of UV–visible radiation by the NCs (Fig. 3).

### Antibacterial testing

**Fig. 4:**
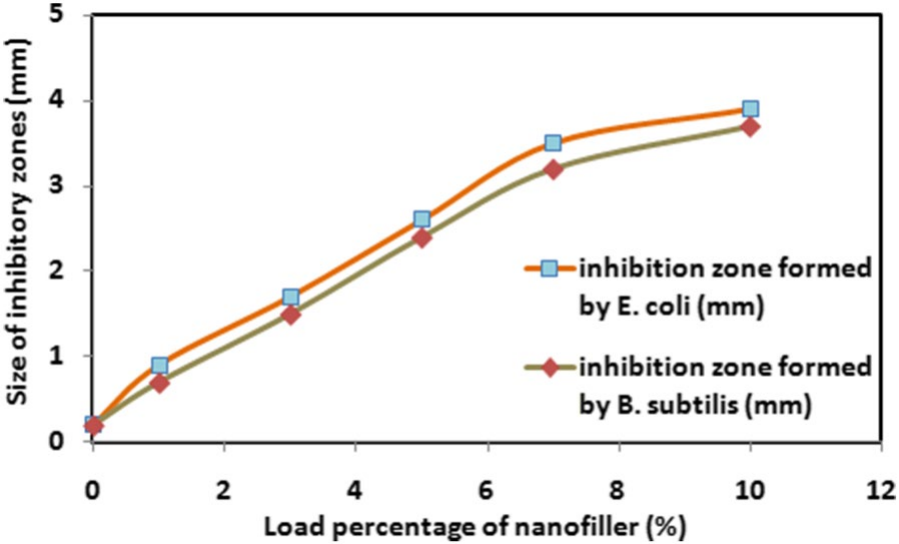
Size of inhibitory zones versus load percentage of Cr in nanocomposites

The POC and composites prepared were evaluated against different strains of gram-positive and gram-negative bacteria. The results showed that POC/Cr NCs were significantly inhibited the bacterial population analogous to other reported materials. The antibacterial activity of the composites was determined against *E. coli* and *B. subtilis* and compared with POC as plotted in Fig. 4. The antibacterial test was carried out by standard agar disc diffusion method [24].

The composites exhibited better antibacterial potential than POC as evident from Table 1. The antibacterial activity was amplified with the increase in the load percentage of the Cr nanofiller. The size of inhibitory zones was measured against two bacterial strains and it is found that the size around POC was minimized. The antibacterial potential was correlated with load percentage of the nanofiller and size of inhibition zone was maximized in case of POC/Cr NCs containing 10% Cr nanofiller. The reason was the availability of maximum inhibitory nanoparticles to counter the growth of bacteria. It is evident that composites were equally effective against both gram-positive and gram-negative bacteria.

**Table 1:** Size of inhibitory zones of antibacterial test of the POC/Cr nanocomposites

Load percentage of nanofiller (%)	Size of inhibitory zones (mm)	
	*E. coli*	*B. subtilis*
1%	0.9	0.7
3%	1.7	1.5
5%	2.6	2.4
7%	3.5	3.2
10%	3.9	3.7
POC	0.2	0.2

### SEM images of POC and POC/Cr NCs

**Fig. 5:**
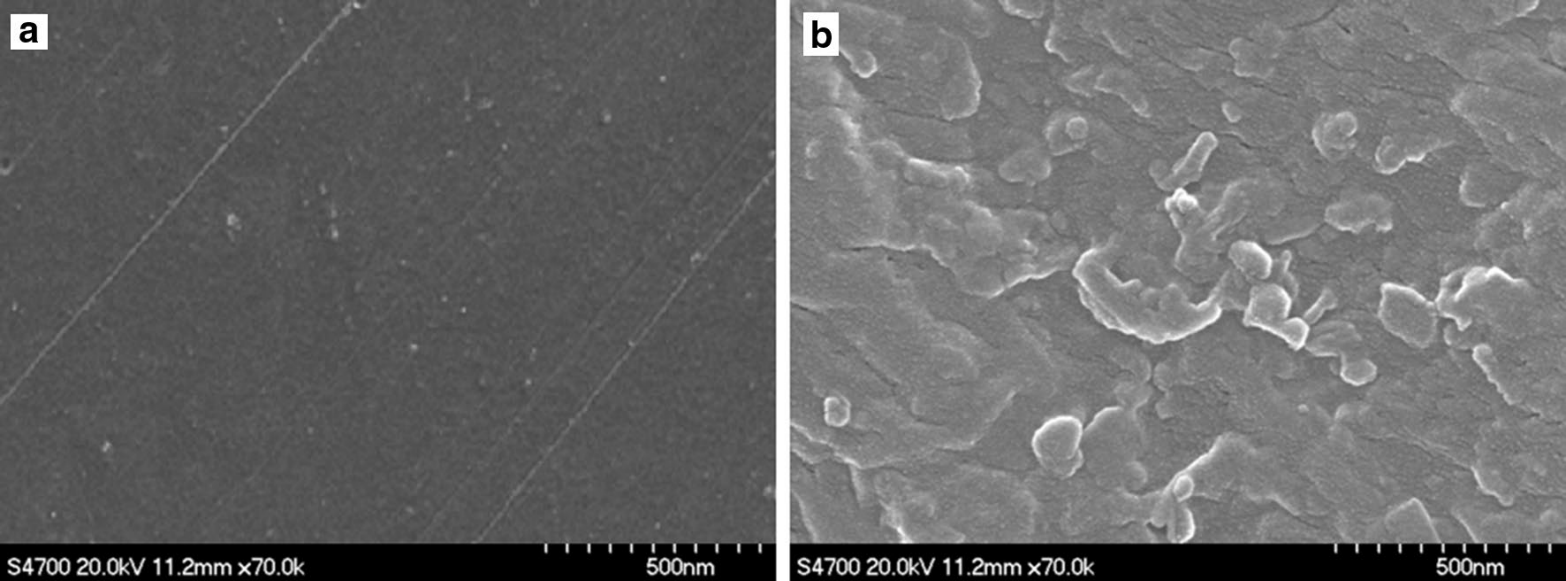
SEM images of **a** POC and **b** POC/Cr NCs containing 7% Cr NPs

SEM micrographs of POC and POC/Cr NCs were recorded to study the morphology of the materials. The SEM images were displayed which illustrated the compactness and grainy structure of POC/Cr NCs due to the presence of nanofiller. The results exhibited the uniform dispersion of nanofiller in the polymer. The POC/Cr NCs containing 7% NPs was represented and explained the composite morphology of the materials. The SEM images also showed that chromium nanoparticles are fully embedded in the polymer matrix and nanocomposite became increasingly compact. Chromium nanoparticles also dispersed uniformly in the polymer although at a meager agglomeration of nanoparticles and illustrated the grainy morphology for all the polymer nanocomposites [23] (Fig. 5).

### Electrical conductivity measurement

The conductivity of POC/Cr NCs was recorded and compared with POC as a reference. The electrical conductivity was measured by dispersion method at room temperature (25 °C) in S/cm unit. The electrical conductivities of POC and POC/Cr NCs were given in Table 2 [25].

**Table 2:** Electrical conductivity of POC/Cr NCs

Samples	Electrical conductivity^a^ (S/cm)
1%	103
3%	114
5%	150
7%	240
10%	350
POC	3

^a^ At room temperature

**Fig. 6:**
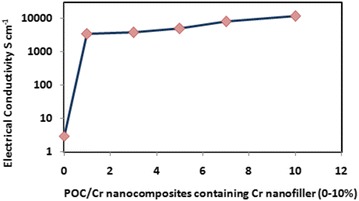
Plot of electrical conductivity versus load percentage of Cr NPs in nanocomposites

With the increase in the load percentage of Cr nanofiller, the conductivity was also increased. The reason is that formation of polarons in POC/Cr nanocomposite. The increase in conductivity values was due to the interaction of conducting polymer matrix with neighboring nanoparticles [11]. The increase in conductivity POC/Cr NCs graphically was represented in Fig. 6. It is evident from conductivity data, the increase in conductivity was due to the contribution of chromium nanoparticles that were embedded in POC matrix. In fact, the POC/Cr composite containing 1% Cr NPs showed significant improvement in the electrical conductivity compared with POC. The higher percentages of Cr NPs (3–10%) were added just to evaluate the improvement behavior in the conductivity of the composites. The composites containing higher percentages of Cr NPs (3–10%) showed slight improvement in the conductivity due to the agglomeration of the Cr NPs. The heavy loading of Cr NPs resulting in slight improvement could not be justified. Therefore, the minimum loading of Cr NPs (1%) not only avoids the agglomeration but also makes the material cost effective.

## Conclusions

In summary, synthesis of POC was carried out by oxidative polymerization of *o*-chloroaniline using ammonium sulfate as oxidizing agent and HCl as a doping agent. Further, POC was composited with Cr NPs by varying the load percentage of nanofiller (1–10%). Different characterization techniques were employed to confirm the structure and properties of the nanocomposites. FTIR studies elucidated the structural features, UV–visible data demonstrated the absorption properties, and SEM imaging demonstrated the morphology of the NCs. The composites containing 10% Cr nanofiller exhibited maximum efficacy against *E. coli* and *B. subtilis.* The electrical conductivity measurements of POC/Cr NCs were recorded by dispersion method. The electrical conductivity values of composites containing nanofiller were better than POC. The composites formed could find the potential applications as antibacterial and conducting materials for biological and electronic devices.

## Abbreviations

Cr: chromium
POC/Cr NCs: polyorthochloroaniline/chromium nanocomposites
NPs: nanoparticles
POC: polyorthochloroaniline
SEM: scanning electron microscopy
FTIR: fourier transform infrared spectroscopy
UV–visible: ultraviolet visible
PANI: polyaniline
DDPA: 4,4′-diaminodiphenylamine
APS: ammonium persulfate
*E. coli*: *Escherichia coli*
*B. subtilis*: *Bacillus subtilis*
RPS: round per minute
KBr: potassium bromide
1 M: 1 molar
HCl: hydrochloric acid
NH_4_OH: ammonium hydroxide
